# The Reliability of PCL/Anti-VEGF Electrospun Scaffolds to Support Limbal Stem Cells for Corneal Repair

**DOI:** 10.3390/polym15122663

**Published:** 2023-06-13

**Authors:** Emilija Zdraveva, Tamara Dolenec, Mirna Tominac Trcin, Emi Govorčin Bajsić, Tamara Holjevac Grgurić, Antoneta Tomljenović, Iva Dekaris, Josip Jelić, Budimir Mijovic

**Affiliations:** 1 Faculty of Textile Technology, University of Zagreb, 10000 Zagreb, Croatia; antoneta.tomljenovic@ttf.unizg.hr (A.T.); josip.jelic@ttf.unizg.hr (J.J.); 2 Department of Transfusion and Regenerative Medicine, Sestre Milosrdnice University Hospital Center, 10000 Zagreb, Croatia; tamara.dolenec@kbcsm.hr; 3 The Institute of Immunology, 10000 Zagreb, Croatia; mirna.tomtrcin@gmail.com; 4 Faculty of Chemical Engineering and Technology, University of Zagreb, 10000 Zagreb, Croatia; egovor@fkit.hr; 5 School of Medicine, Catholic University of Croatia, 10000 Zagreb, Croatia; tamara.holjevac.grguric@unicath.hr; 6 Faculty of Medicine, University of Rijeka, Bilić Vision Polyclinic, 10000 Zagreb, Croatia; iva.dekaris@gmail.com

**Keywords:** electrospinning, PCL, anti-VEGF, scaffolds, LSCs, physicochemical performance, immunocytochemistry

## Abstract

Since only few reported studies propose anti-vascular endothelial growth factor (anti-VEGF) delivery through electrospun scaffolds, this study greatly contributes to the potential prevention of patient’s vision loss, as it explores electrospun polycaprolactone (PCL) coated with anti-VEGF for the blockage of abnormal cornea vascularization. In terms of physicochemical properties, the biological component increased the PCL scaffold fiber diameter (by ~24%) and pore area (by ~82%), while ut slightly reduced its total porosity as the anti-VEGF solution filled the voids of the microfibrous structure. The addition of the anti-VEGF increased the scaffold stiffness almost three-fold at both strains of 5 and 10%, as well as its biodegradation rate (~36% after 60 days) with a sustained release profile after Day 4 of phosphate buffered saline incubation. In terms of scaffold application function, the PCL/Anti-VEGF scaffold proved to be more favorable for the adhesion of cultured limbal stem cells (LSCs); this was confirmed by the SEM images, where the cells showed flat and elongated conformations. Further support of the LSC growth and proliferation was confirmed by the identified p63 and CK3 markers after cell staining. These results demonstrate the advantageous effect of the surface-adsorbed anti-VEGF to stop vision loss and help damaged corneal tissue repair.

## 1. Introduction

Corneal neovascularization (NV) is a pathological condition that causes excessive blood vessel ingrowth from the limbal zone to the stroma, with time resulting in the reduction of the eye visual acuity. Diseases associated with this condition include: trauma (i.e., chemical burns), inflammation (i.e., corneal graft rejection, atopic conjunctivitis), infection (fungal, bacterial, viral), degenerative conditions, congenital disease (i.e., limbal stem cell deficiency, LSCD), and so forth [1,2]. Vascular endothelial growth factor (VEGF) is a protein that promotes new blood vessel growth (angiogenesis) in case of i.e., eye inflammation after injury [3]. The anti-vascular endothelial growth factor (anti-VEGF), on the other hand, is a medical agent used to block the function of the VEGF [4], which is why it is of great importance in controlling corneal NV. The anti-VEGF is usually injected directly into the vitreous (back) cavity of the eye (so called intravitreal drug delivery). Unfortunately, this type of drug administration is the cause of many serious complications, including infectious endophthalmitis, intraocular inflammation, rhegmatogenous retinal detachment, intraocular pressure elevation, ocular hemorrhage, or rare ocular, and systemic side effects [5]. Some of the limitations in the anti-VEGF topical administration include the physical structure of the cornea, the vitreous, blood vessels, and the tear film, all slowing down the drug delivery to the retina [5,6]. Due to the aforementioned issues, the application of this biological component requires special attention in terms of the design of a target sustained drug-delivery system. As reported, the anti-VEGF drug-delivery system needs to fulfill the following requirements: biocompatibility, sustained and target delivery over one month, transparency, adequate concentration, and bioactivity [7]. An innovative approach in the design of this type of drug-delivery systems in recent years is the technique of electrospinning [8], where the system is a nanofibrous material. The technique of electrospinning uses an electrostatic force to stretch a viscoelastic polymer solution for the fabrication of nanofibers (or microfibers). In the field of nanomedical applications, the design of drug-delivery systems garners special attention since this technique successfully encapsulates medical or biological species in the nano-fibrous/micro-fibrous materials functionalized for fast, sustained, or tunable drug release. At the same time, these structures sufficiently support specific cell-type cultures and can help in the regeneration of damaged tissues by promoting their adhesion, migration, and proliferation [9]. Recently reported studies concerned with the fabrication of electrospun fibrous scaffolds mainly incorporate VEGF or (in a few studies) anti-VEGF compounds, generally for tissue-engineering applications. Electrospun polycaprolactone (PCL) was plasma treated to immobilize VEGF by covalent bonding. Human umbilical vein endothelial cells (HUVECs) were cultured on the scaffolds, and it was observed that the biological activity of the immobilized VEGF induced higher cell numbers after 9 days of culture due to its sustained action [10]. VEGF was also loaded into core/shell electrospun dextran (DEX)/poly(lactide-co-glycolide) (PLGA) fibrous scaffolds for vascular tissue regeneration. The scaffolds showed sustained release over a period of 28 days, while the VEGF promoted HUVECs proliferation, spreading cell–membrane interactions [11]. Co-electrospinning was also conducted for the fabrication of PCL/gelatin scaffolds. Scaffold-surface immobilization with VEGF was further carried after gelatin functionalization with heparin. Significantly improved endothelial cell proliferation was observed over the sustained release of the VEGF for 25 days. Excellent vascularization after 4 weeks of subcutaneous implantation of the scaffolds in rats was confirmed by almost full cellularization of the same [12]. Electrospun polyurethane (PU) scaffolds were loaded with polyethylene glycol (PEG)-coated cerium oxide nanoparticles (CNPs) and VEGF to be used as small-diameter artificial blood vessels for endothelialization in vitro. The scaffolds were cultured with endothelial progenitor cells (EPCs). The scaffolds were designed to obtain a synergistic effect of both the VEGF and the CNPs, i.e., the effect on angiogenesis and anti-EPCs apoptosis effect, respectively. The release of the CNPs and VEGF was sustained over 30 days. The scaffolds inhibited H_2_O_2_-induced EPCs apoptosis, while the CNP–PEG enhanced VEGF activity to support mobilization and differentiation of the EPCs [13]. Scaffolds with orthogonally aligned poly(lactic-co-glycolic acid) (PLGA) nanofibers and controlled dual release of VEGF and platelet-derived growth factor (PDGF) were fabricated by emulsion electrospinning. The scaffolds successfully directed and stimulated the function of HUVECs and human aortic smooth muscle cells to help in early-stage vascularization and smooth muscle maturation [14]. VEGF is also known for its role in bone osteoblast growth and mineralization [15]. Three-dimensional-printed hydroxyapatite/calcium sulfate/PCL scaffolds were loaded with VEGF to be utilized in bone-tissue engineering. In-vitro results showed enhanced human mesenchymal stem cells (hMSCs) and HUVEC proliferation, while in-vivo studies resulted in rabbit’s femur bone regeneration after 8 weeks of implantation [16]. The frequent invasive dosing of anti-VEGF in case of age-related macular degeneration treatment was reported to be replaced by electrospun core/shell bevacizumab/PCL system with a constant rate of release of the medicine over 2 months [8]. Anti-bone morphogenic protein 2 (anti-BMP-2) and anti-VEGF were immobilized on the surface (separate areas) of UV-ozone irradiation-activated electrospun PCL with a parallel pattern design. Human bone marrow-derived mesenchymal stem cells were positive on osteogenic and angiogenic markers in areas where the BMP-2 and VEGF were bound, respectively. A significantly increased angiogenic response was obtained in an in-vivo chick chorioallantoic membrane (CAM) assay. Thus, the scaffolds have confirmed their role in guided bone regeneration, i.e., for internal bone fixtures or flat bone healing [17]. Other techniques used in the encapsulation of both VEGF and anti-VEGF compounds (except electrospinning and 3D printing) included the gas-foaming process, employed for a simultaneous stable release of the VEGF and a dose-dependent release of the anti-VEGF agent [18]. Freeze-drying [19] and hydrogelling [20], as well as colloidal crystal templating [21], were reported for the fabrication of hydroxyapatite-collagen scaffold loaded with VEGF, collagen/gold nanoparticles scaffold incorporating anti-VEGF, and 3D porous polydimethylsiloxane (PDMS) scaffold for a 30-month delivery of anti-VEGF, respectively. In this study, electrospun PCL was incorporated with an anti-VEGF agent via a post-processing wetting treatment. The PCL/anti-VEGF scaffolds were evaluated for their physicochemical properties and final role in the support of limbal stem cells (LSCs) to be used in corneal tissue engineering. To the best of our knowledge, the majority of reported studies explore electrospun scaffolds for the delivery of the aforementioned VEGF agent. Thus, the current study greatly contributes to the earlier discussed topic in both effects of the anti-VEGF agent on the scaffold properties and cultured cells.

## 2. Materials and Methods

The polymer used as the matrix in this study was polycaprolactone (PCL) with Mn = 80,000 (Sigma–Aldrich, Burlington, MA, USA). The solvents used were glacial acetic acid and acetone (Ru–Ve). Anti-vascular endothelial growth factor (anti-VEGF) solution (25 mg/mL bevacizumab, Avastin, Roche) was kindly supplied by Bilić Vision Polyclinic, Zagreb, Croatia. Eighteen percent of PCL solution concentration was prepared by dissolving the polymer in the glacial acetic acid and acetone mixture, with the volume ratio of 8:2, respectively, under constant stirring for more than 24 h.

**Figure 1:**
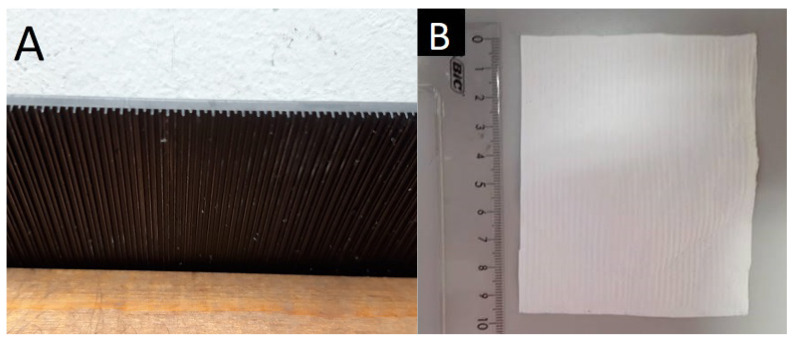
Pattern electrospinning; (**A**) 3D-printed collector with ribbed geometry, (**B**) Electrospun PCL scaffold.

Single electrospun PCL was fabricated with the electrospinning device NT–ESS–300, NTSEE Co. Ltd., Gwangju, Republic of Korea, on a ribbed 3D-printed collector (Form 2 3D printer FormLabs, Somerville, MA, USA) treated with a graphite coating, Figure 1A. The dimensions of the collector ribs were the following: height of 1.2/0.6 mm, width of 0.3 mm, and rib distance of 0.8 mm. The processing conditions were electrical voltage of 14–20 kV, needle tip to collector distance of 18 cm, and volume flow rate of 1 mL/h. The environmental conditions were temperature of 20 °C and humidity of 40%. The electrospun PCL scaffolds, Figure 1B, were soaked in the 25 mg/mL anti-VEGF solution post-processing for 2 h and dried for 24 h at room temperature afterwards.

### 2.1. Evaluation of the Scaffolds’ Physicochemical Performance

Attenuated total reflectance–Fourier transform infrared (ATR–FTIR) spectroscopy Perkin Elmer Spectrum One was used to confirm the presence of the anti-VEGF on the surface of the electrospun scaffold. The measurements were carried out at room temperature in the wavelength range of 4000 to 650 cm^−1^.

The morphology of the PCL and PCL/anti-VEGF electrospun scaffolds was analyzed by scanning electron microscopy Jeol, JSM 7000F (Tokyo, Japan) and Tescan Vega TS 5136 MM (Brno, Czech Republic). The scaffold’s fiber diameter and pore area were determined based on the SEM images by measuring 100 randomly selected fibers or pores, respectively, using the ImageJ–NIH software. The porosity was calculated according to the equation provided elsewhere [22] based on scaffolds’ thickness, weight, area, polymer, and blend densities. The thickness of the scaffolds was measured by a Digi Micrometer, Mitutoyo, Kawasaki, Japan.

The water contact angle was measured based on the images (Dino Capture 2.0 microscopy) of a water droplet (volume of 0.5 mL) placed on the surface of the scaffolds by using the Low Bond–Axisymmetric Drop Shape Analysis (LB–ADSA) tool in the ImageJ software [23]. The wettability was evaluated at 0 and 3–5 s after drop placement.

The scaffolds were tested under tensile load with Tensile Strength Tester Tensolab 3000, Mesdan S.p.A., Puegnago Del Garda, Italy. The load cell was 100 N, the gauge length was 20 mm, and the rate of extension was 20 mm/min. The dimensions of the samples were 10 × 90 mm.

Dynamic mechanical analysis was conducted on DMA 983, TA Instruments with the following testing conditions: frequency of 1 Hz, amplitude of 0.2 mm, heating rate of 3 °C/min, and a temperature range between −100 °C and 100 °C. The cooling was conducted under liquid nitrogen.

The release profile of the biological component, the anti-VEGF, was determined by monitoring the behavior of the electrospun 18% PCL/anti-VEGF scaffold (dimensions of 20 × 20 mm) incubated in 10 mL of PBS (phosphate buffered saline) solution at 37 °C (with continuous stirring at 80 rpm) for a period of 7 days. A portion (2–3 mL) of the solution was withdrawn every 24 h to determine the absorbance by the UV–Vis transmission spectrophotometer Cary 50, Varian. The solutions were returned to the bulk to maintain the same volume during the testing period. The concentrations of the released biological components were calculated based on a standard curve obtained from known concentrations (from 0.13 to 0.5 mg/mL) of the anti-VEGF in the PBS.

The biodegradability of the PCL and the PCL/anti-VEGF electrospun scaffolds was examined through incubation in PBS solution for 7 to 60 days at a temperature of 37 °C. The weight loss of the scaffolds was calculated based on the initial dry mass and the dry mass of the samples after incubation [24]. It is known that the degradation rate of the PCL is very slow, and it usually takes from at least 6 months up to 4 years [25]. Therefore, the degradability of the scaffolds during the period envisaged as the therapy period (up to 14 days) can be attributed mostly to the release of the bioactive component. All the aforementioned tests were carried out in triplicates.

### 2.2. Human LSCs Isolation, Culture and Immunocytochemistry

Detailed procedure of the human LSC cultivation and immunocytochemical analysis was described in our previous study [26]. Briefly, the first step is the preparation of the 3T3 (mouse cells, ATCC-CCL-92, Swiss albino) feeder layer in a growth medium containing Dulbecco’s Modified Eagle Medium (DMEM) (Gibco, Invitrogen, Waltham, MA, USA), 10% of heat inactivated Fetal Bovine Serum (FBS) (Gibco, Invitrogen, Waltham, MA, USA), antibiotic-antimycotic (ABAM), and 1% L-glutamine. Further proliferation of the trypsinized 3T3s was inhibited with the FBS medium and γ-rays. The second step involves the isolation of the LSCs from surgical remains of human cadaveric cornea with permission of the Ethics Committee of the University Eye Hospital Svjetlost (Zagreb, Croatia). Previously cut (15 mm in diameter) and disinfected (under UV-light) scaffolds were seeded with the 3T3s and LSCs on top in 24 well plates with 1.5 × 10^4^ cells/cm^2^ per well with the ratio of 1:1. The LSCs were grown until 70–100% of confluence with the change of the keratinocyte growth medium every third day.

Indirect immunocytochemistry (ICC) first involved fixation of the cells with 4% paraformaldehyde (Sigma–Aldrich, Burlington, MA, USA), permeabilization with 0.5% triton X-100 (Sigma–Aldrich, Burlington, MA, USA), and antibody incubation. The primary and secondary antibodies used were the mouse monoclonal antibody [4A4] IgG2a to human p63 protein (Abcam, Cambridge, UK), and mouse monoclonal antibody [AE5] IgG1 to human cytokeratin 3/CK-3 (Abcam, Cambridge, UK), as well as goat anti-mouse IgG Alexa Fluor 488 (Invitrogen, Thermo Fisher Scientific, Waltham, MA, USA) diluted in phalloidin-tetramethylrhodamine B isothiocyanate TRITC (Sigma–Aldrich, St. Louis, MO, USA) and goat anti-mouse IgG Alexa Fluor 568 (Invitrogen Thermo Fisher Scientific, Waltham, MA, USA), respectively. Cell nuclei were marked with 4′,6-diamidino-2-phenylindole (DAPI) (Sigma–Aldrich, Burlington, MA, USA) and imaged under confocal microscopy Leica, TCS SP2 AOBS (Leica Microsystems CMS GmbH, Mannheim, Germany) at the Ruder Bošković Institute in Zagreb, Croatia.

## 3. Results

**Figure 2:**
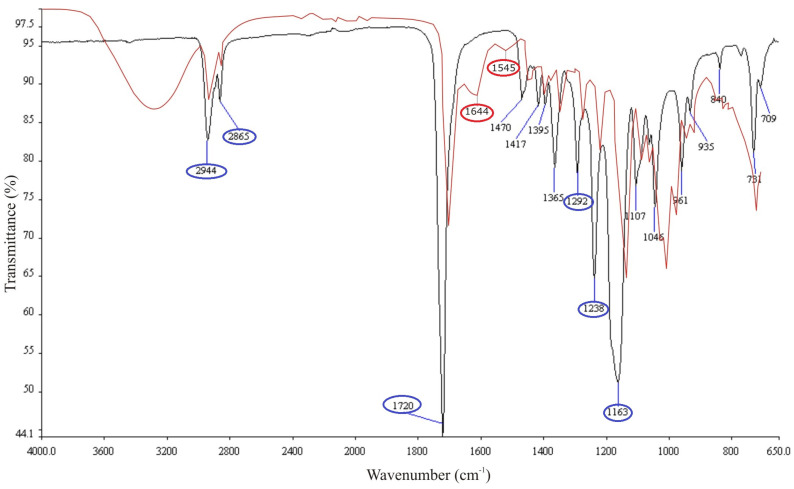
FTIR transmission spectra of the electrospun PCL (black line) and PCL/Anti-VEGF (red line) scaffolds.

Figure 2 shows the FTIR transmission spectra of the electrospun single PCL and anti-VEGF-coated PCL scaffolds. The analysis was conducted to confirm the anti-VEGF coating on the surface of the electrospun PCL scaffold. The characteristic functional groups for the single PCL (black line) are at the wavelengths of 2944 cm^−1^ and 2865 cm^−1^, which corresponds to the –CH_2_– group. Further, the amorphous phase of the PCL is presented by the carbonyl group C=O at the wavelength of 1720 cm^−1^, while the band at 1292 cm^−1^ corresponds to the C–C and C–O bonds in the crystalline phase of the PCL. The absorption bands at 1238 cm^−1^ and 1163 cm^−1^ correspond to the asymmetric and symmetric C–O–C bond stretching, respectively [27]. The anti-VEGF coating (red line) showed many vibrational frequencies, but the most relevant to the protein molecule are at the wavelength of 1644 cm^−1^ related to Amide I, originated from C=O stretching, and at the wavelength of 1545 cm^−1^ related to Amide II band [28].

**Figure 3:**
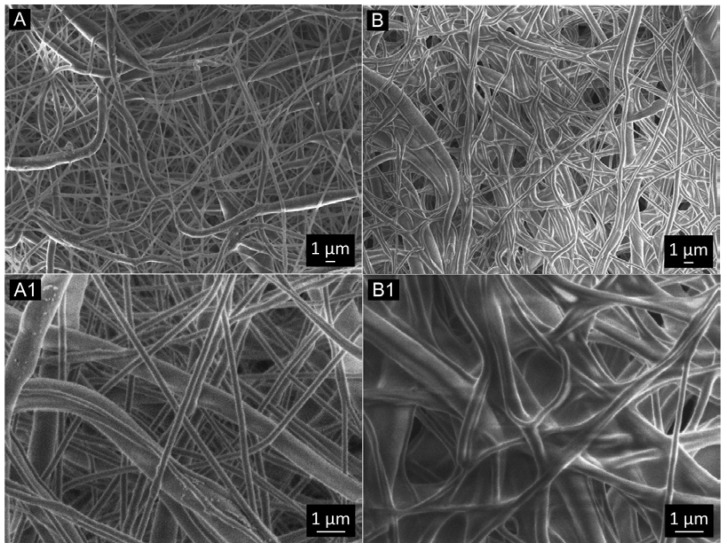
SEM photomicrographs of the electrospun scaffolds: (**A**,**A1**) PCL and (**B**,**B1**) PCL/Anti-VEGF.

Figure 3 shows the SEM images of the electrospun single PCL; Figure 3A,A1 and anti-VEGF coated PCL scaffolds; Figure 3B,B1. Generally, the fibers showed a homogeneous appearance with smooth surfaces, random deformations (beads), and a few thicker fibers on the scaffold surfaces. The anti-VEGF coating filled the pores in between the fibers, while some of the fibers observed showed a swollen appearance due to anti-VEGF solution adsorption, Figure 3B1.

**Figure 4:**
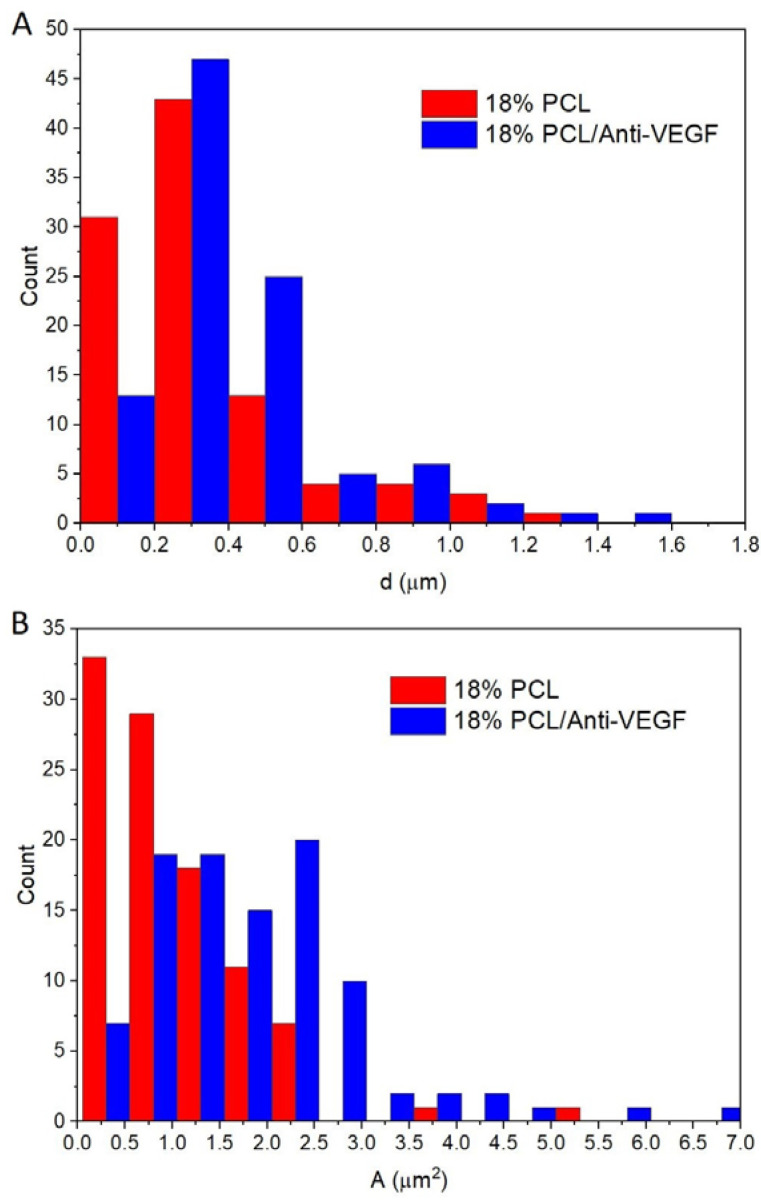
(**A**) Fiber diameter and (**B**) pore-area distribution of the electrospun PCL and PCL/anti-VEGF scaffolds.

Figure 4 shows the distributions (histograms) of the electrospun scaffolds’ fiber diameter, Figure 4A, and pore area, Figure 4B. The ranges of diameters and areas were almost fully overlapping for the electrospun scaffolds. The mean fiber diameters of the electrospun PCL and PCL/anti-VEGF scaffolds were 0.342 ± 0.252 μm and 0.424 ± 0.260 μm, respectively, while the mean pore areas were 1.006 ± 0.812 μm^2^ and 1.829 ± 1.170 μm, respectively.

ANOVA one-way and Tukey tests revealed that the mean values of the fiber diameter and pore areas are significantly different at the level of 0.05 between the PCL and the PCL/anti-VEGF electrospun scaffolds. The total porosity of the electrospun single PCL was calculated to be almost 85% (84.60 ± 9.93), while that of the scaffolds with the added biological component were calculated to be about 75% (75.43 ± 9.13). Statistically, there was no significant difference between the two means of electrospun scaffolds.

**Figure 5:**
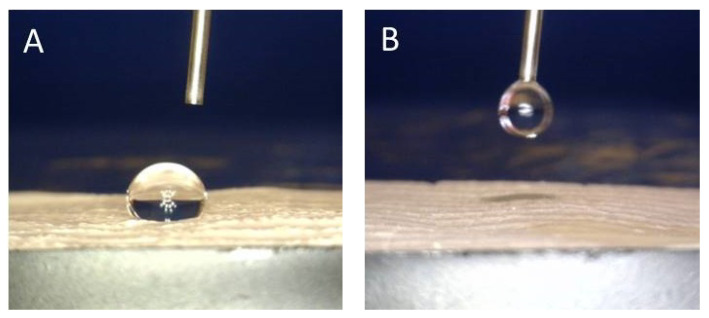
Water contact angle measurement on the surface of the electrospun scaffolds: (**A**) PCL and (**B**) PCL/anti-VEGF.

Figure 5 shows the wetting ability on the surface of the electrospun scaffolds. The hydrophobic nature of the electrospun single PCL scaffold is confirmed by the large water contact angle measured (122.18 ± 1.80°), which does not change even after a few seconds, Figure 5A. On the other hand, the addition of the anti-VEGF results in high scaffolds’ hydrophilicity or immediate adsorption of the drop of water on the scaffolds’ surface, Figure 5B.

**Figure 6:**
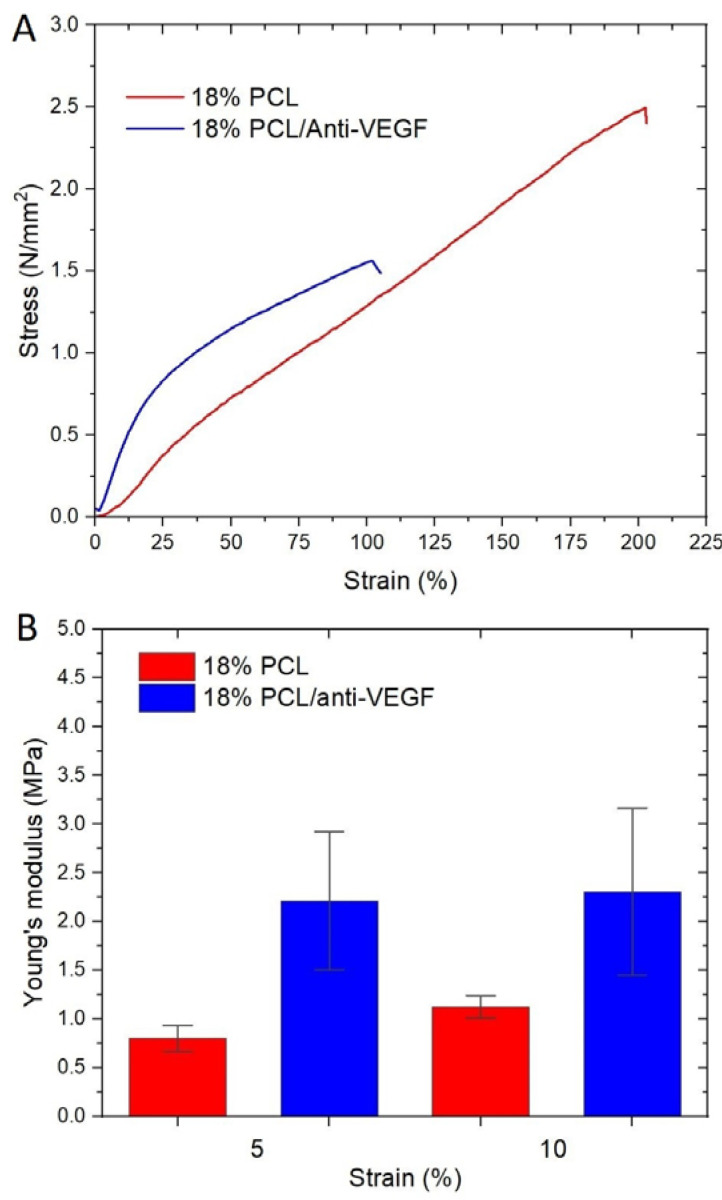
(**A**) Tensile stress–strain curves and (**B**) Young moduli of the electrospun scaffolds.

Figure 6 shows the stress–strain curves, and, Figure 6A, those of the electrospun scaffolds after tensile testing. The calculated Young moduli at the strain of 5 and 10% are shown in Figure 6B. The aforementioned strain values were determined as the elastic region limit values.

Table 1 shows the maximum force, elongation at break, and tensile strength of the electrospun scaffolds.

The addition of the anti-VEGF has evidently reduced the stretching ability of the PCL scaffolds from 181.85 ± 21.65 to 99.00 ± 18.19%, as well as the maximum tensile strength from 2.31 ± 0.18 to 1.41 ± 0.14 N/mm^2^. At both strain values (5 and 10%), the electrospun PCL/anti-VEGF scaffold shows a significantly higher elastic modulus (from 0.80 ± 0.27 to 2.21 ± 1.41 MPa, and from 1.12 ± 0.23 to 2.73 ± 1.39 MPa), although after the strain of 10%, the scaffold behaves plastically, which is not the case with the single PCL. The results indicate that the addition of the biological component directly affects the elasticity of the scaffolds.

**Figure 7:**
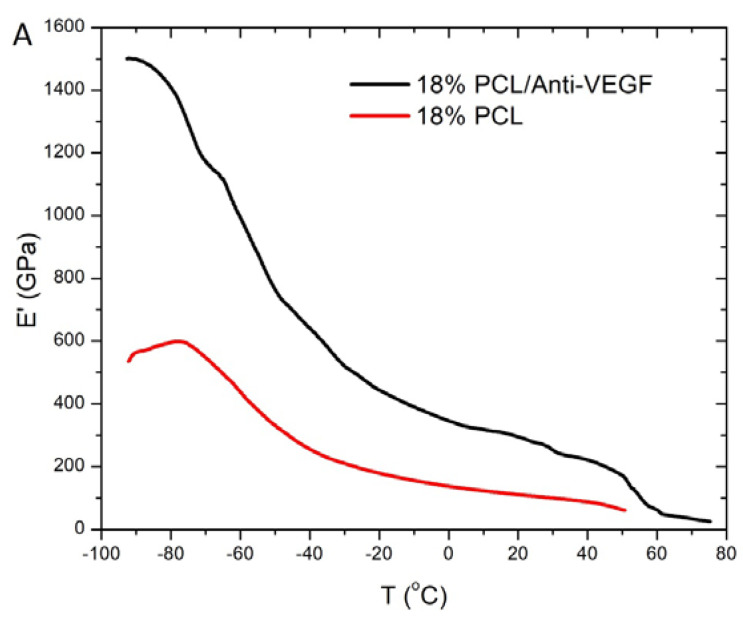

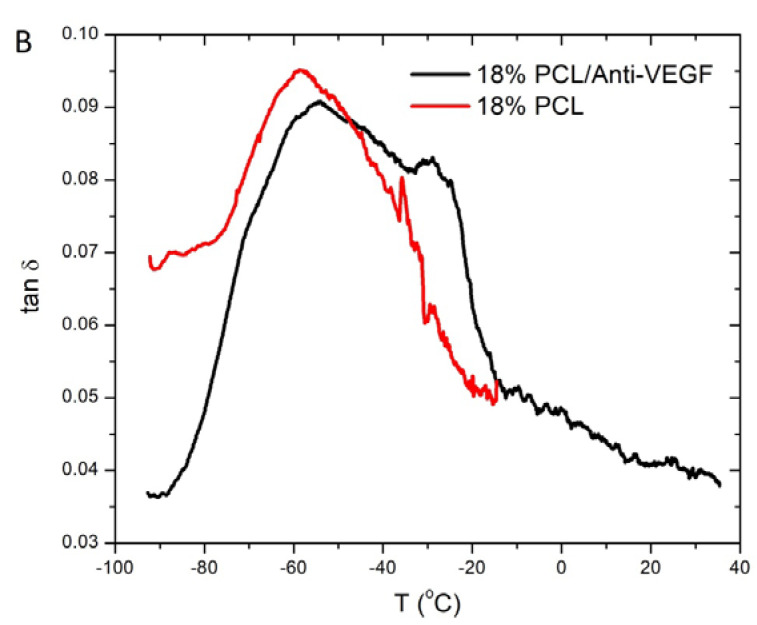
DMA analysis: (**A**) Storage modulus as a function of temperature and (**B**) Damping as a function of temperature of the electrospun scaffolds.

In terms of the anti-VEGF effect, the mechanical behavior of the electrospun scaffolds was further analyzed to determine the viscoelastic properties of the electrospun scaffolds, including storage modulus, E’, and loss tangent tanδ as a function of temperature, as shown in Figure 7A,B, respectively.

The storage modulus curve is typical for the semi-crystalline PCL polymer. A smaller drop in the storage modulus can be observed below the glass transition temperature (T_g_) of −60 °C. While above it, a sharp decrease in the storage modulus is recorded, which corresponds to the amorphous phase of the polymer. Up to a temperature of 40 °C, the drop in the storage modulus is due to the progressive melting of the polymer. While above a temperature of 50–60 °C, plastic deformations occur due to the complete melting of the crystalline phase [29]. A significant increase in the storage modulus is observed with the addition of the anti-VEGF, which indicates an improvement in the mechanical behavior of the scaffold due to the strengthening effect of the biological component. The relaxation maxima at temperatures below zero corresponds to the glass transition temperature of the amorphous phase of the PCL. The addition of the anti-VEGF results in a shift of the T_g_ to higher temperatures (−55 °C), compared to the single electrospun PCL scaffold (−60 °C). The results indicate the placement of the particles of the anti-VEGF in the amorphous phase of the PCL and the reduction of its mobility.

**Figure 8:**
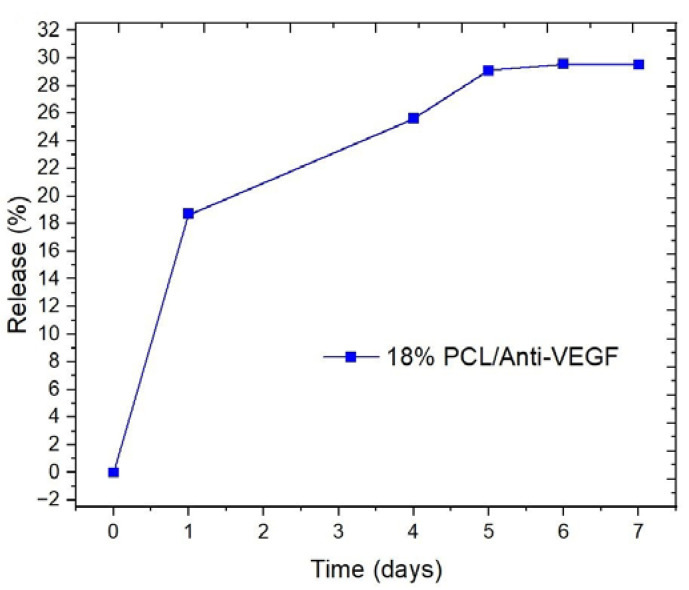
Anti-VEGF release profile from the electrospun PCL/anti-VEGF scaffold.

The release profile of the anti-VEGF in the PBS solution (Figure 8) is determined based on the standard curve concentrations of the biological component (from 0.13 to 0.5 mg/mL) in the PBS. The release profile includes two phases: 1. Immediate release of the biological component of about 19%, with a maximum of 30% of the anti-VEGF (from Day 1 to Day 5), and 2. Continuous release of the remaining amount of the anti-VEGF, which means a relatively constant concentration of the biological component. The described profile and calculated concentrations correspond to a period of 7 days, after which it was not possible to determine the maximum absorbance peak. In the period from 12 to 14 days, a slightly smaller peak appeared (not given here), i.e., the absorbance was read at another wavelength of 760 nm, which can be assumed to correspond to the degradation signal of the biological component. The largest amount of the anti-VEGF component was released in the first 24 h due to its placement on the surface of the electrospun PCL scaffold, which is the result of the post-processing treatment.

The release profile is based on the diffusion of the encapsulated anti-VEGF component, which slows down over time. The slow release is the result of the hydrophobic PCL matrix, taking more time to absorb the PBS solution during incubation.

Table 2 shows the percentage of weight loss for the electrospun scaffolds incubated in the PBS solution for the period of 7 to 60 days. The weight loss of the electrospun PCL/anti-VEGF showed a gradual increase with an initial percentage loss of 14% to almost 36% after 60 days of incubation. This behavior of the scaffolds favors the healing process in corneal therapy, i.e., it enables blockage of the vascularization of the visual eye field during the regeneration of the eye tissue. A negative percentage of the weight loss was observed in cases of the electrospun single PCL scaffold, which indicates that no degradation of the scaffold occurs after the total time of incubation. Certainly, the addition of the active biological component improves the degradability of the scaffold, which is in favor of eye therapy.

**Figure 9:**
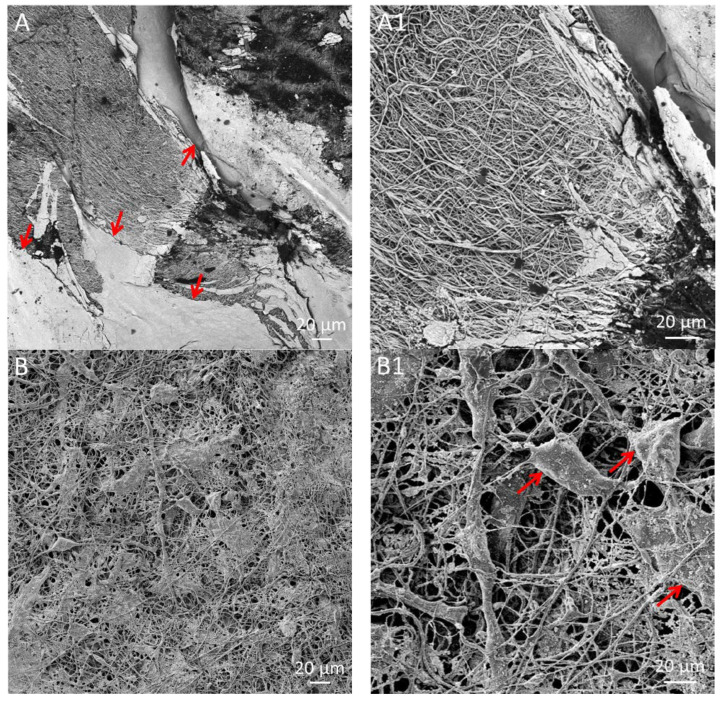
SEM photomicrographs of the adhered LSCs on the electrospun scaffolds: (**A**,**A1**) PCL and (**B**,**B1**) PCL/anti-VEGF.

Figure 9 shows representative SEM photomicrographs of the electrospun PCL (Figure 9A,A1), and PCL/anti-VEGF scaffolds after LSC cultivation (Figure 9B,B1). The scaffolds showed partial or full coverage of their surface by the adhered LSCs, with a more favorable cell adherence in case of the hydrophilic scaffold surface of the PCL/anti-VEGF system. The shapes of the LSCs were more clearly observed in case of the PCL/anti-VEGF scaffolds, where most of the cells showed flat and elongated conformations (Figure 9B). The cells were mostly joint and filling the voids in between the fibers.

**Figure 10:**
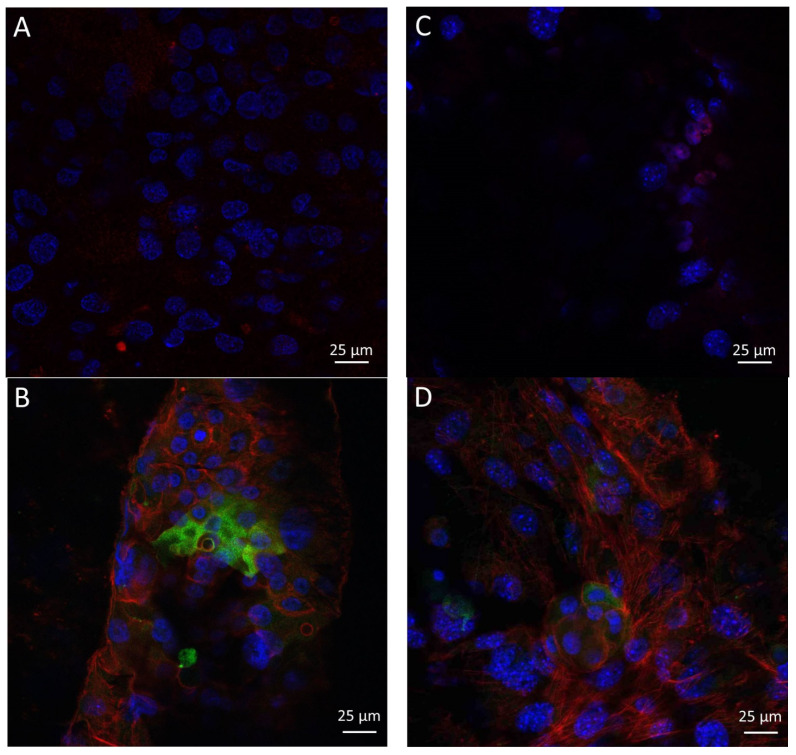
Immunofluorescence analysis of the limbal stem cells cultured on: (**A**,**B**) PCL and (**C**,**D**) PCL/anti-VEGF scaffolds. Cells positive on cornea marker CK3 have cytoplasm colored red (**A**,**C**) and cells positive on stem cell marker p63 show nuclei stained with turquoise. Cytoskeleton is stained red with phalloidin TRITC (**B**,**D**). All nuclei are counterstained with blue stain 4′,6-diamidino-2-phenylindole (DAPI).

Immunofluorescence analysis of the LSCs cultured on the PCL and PCL/anti-VEGF scaffolds was conducted to evaluate the presence of the limbal stem cell p63 marker, a nuclear transcription factor, and cytokeratin 3 (CK3), which is a corneal epithelial differentiation marker. Figure 10 shows the confocal images of the LSCs on the electrospun PCL (Figure 10A,B), and PCL/anti-VEGF scaffolds after immunofluorescence staining (Figure 10C,D).

The LSCs were identified as positive on cornea marker CK3 when their cytoplasm was colored red, while their nuclei was colored turquoise when the cells were positive on stem cell marker p63. The cytoskeleton of the LSCs was red and all the nuclei were counterstained with DAPI staining. Generally, the confocal images confirmed the observation of the SEM analysis, i.e., both electrospun PCL and PCL/anti-VEGF scaffolds showed support of the LSC adhesion, growth, and proliferation, since both limbal stem cells and differentiated corneal cells were detected on the scaffold surfaces, while the scaffold with the added biological component was expectedly more favorable.

## 4. Discussion

The human cornea is the outermost transparent part of the eye, with a multi-scale structure due to its different layers and fiber pattern organization. The most characteristic architecture includes the one of the Bowman’s layer with its randomly oriented collagen fibrils, while collagen fibrils organized into parallel lamellae and lamellae above one another in the center represent the corneal stroma layer [30,31,32]. The major contribution to the corneal mechanical strength comes from the stroma and the difference in the degree of fibrils alignment and the diameter. Its strength varies with depth as well [33]. The LSCs are situated in the limbus, which is the transition zone between the clear cornea and the opaque sclera (the white of the eye) [34]. The structure of the electrospun PCL/anti-VEGF scaffolds tends to simulate the aforementioned corneal architecture that consists of parallel densely packed fibrous bundles, as well as of randomly loose packed fibers in between. The scaffolds’ structure is a result of the 3D-printed collector ribbed geometry (Figure 1A), as mentioned in earlier sections. This pattern was generated to facilitate cell adhesion and growth. In terms of chemical composition, the addition of the anti-VEGF agent is vital for corneal tissue regeneration, as it prevents vascularization and closure of the eye’s visual field. The evaluation of physicochemical properties of the scaffolds included morphology, porosity, surface wettability, static tensile and dynamic mechanical behavior, biological component release activity, and scaffold degradation ability. Visually, the SEM photomicrographs displayed a swollen appearance (Figure 3B,B1), with the addition of the anti-VEGF agents. This is in accordance with the mean values of the measured fiber diameter, which increased by ~24%. A similar effect was reported in a study of electrospun PLGA fibers treated with plasma, chitosan, and heparin, as well as mineral (hydroxyapatite) coating afterwards, to result in the diameter increase of 200 nm, or up to 1 µm [35]. A significant increase of ~82% in the pore area was also observed after the anti-VEGF coating. This was expected, as the increase in fiber diameter results in increased pore size, as reported in the case of electrospun PCL–hydroxyapatite and PCL–hydroxyapatite/b-tricalcium phosphate with a two-fold increase in fiber diameter and average pore-size area compared to PCL [36]. The increase in pore size usually results in the increase of the scaffolds’ total porosity [37], but an opposite effect was observed in this study, although the difference was not significant statistically (reduction of the porosity after coating for 10.8%). This can be explained through the application of the anti-VEGF solution, which filled the voids in the microporous structure of the scaffolds in depth. The study also reports on the sensitivity of this scaffold’s property to very small changes in the process conditions; thus, the porosity may vary even across the surface of the same electrospun sample [38,39]. Although the overall porosity of the PCL/anti-VEGF scaffold is slightly reduced, it is still high enough (almost 80%) to fulfill the requirements of a tissue-engineering scaffold. High porosity enables the effective release or exchange of bioactive components (i.e., proteins, genes, cells) and nutrients/waste, respectively. At the same time, very high porosity may compromise scaffolds’ mechanical integrity; thus, an optimal value of the same is preferred [40]. Optimal cell adhesion is also possible in case of moderate scaffold hydrophilicity [41], although the addition of the anti-VEGF reduced the water contact angle to zero in this study, whereas other studies reported on the improvement of cell infiltration, proliferation, and differentiation in cases of scaffold high water retention [42]. In terms of scaffold tensile properties, the anti-VEGF coating has reduced the tensile strength of electrospun PCL by ~39%, Table 1, but the single PCL scaffold had a lower strength initially. Generally, lower porosity will increase the scaffolds’ tensile strength [43], but an opposite effect in the current study is in accordance with our previous research in the case of electr-spun PCL/5 wt% Cefuroxime scaffolds [26]. On the other hand, an increase (for almost three-fold at both strains of 5 and 10%) in the scaffold’s stiffness (Young’s modulus, Figure 6B) is observed with the addition of the biological component, which can be explained by the increase in the scaffold’s fiber mean diameter, as reported elsewhere [44,45].

The functionality of the PCL/anti-VEGF scaffold is also evaluated through its release profile (Figure 8). The release pace of the scaffold needs to be in accordance with the patient’s therapy. The initial burst release of the anti-VEGF is explained by the post-processing treatment; i.e., the coating that was mainly present on the fibers’ surfaces [46], was, to a lesser extent, adsorbed inside the fibrous structure. The adsorption rate is affected mainly by the polymer’s high hydrophobicity. After Day 4 of PBS incubation, the PCL/anti-VEGF scaffold showed a sustained release of the bioactive component, which is preferred in retinal disease therapies. It is reported that the physical adsorption of a drug or bioactive component negatively affects their sustained release [47] but, at the same time, maintains their activity or prevents their destabilization and denaturation during the electrospinning process [48]. These results are comparable to a similar study where core-shell electrospun anti-VEGF (Avastin solution)/PCL scaffolds showed biphasic release profiles, beginning with a burst phase over 24 h (total release of ~25% at Day 3), and a further constant rate of release over a period of 19 days (total release of ~60%) [8]. Another study reported on the release of the anti-VEGF compound from core/shell PVA(Anti-VEGF)/PCL (gelatin) scaffolds, showing a smaller released amount and a constant release over a 6-day period, compared to blended electrospun scaffolds [49]. Scaffold biodegradability is another important property among the essential ones required in the process of tissue repair. As expected, the anti-VEGF coating introduced the scaffold with gradual weight loss ability from Day 7 to Day 60, with an almost three-fold increase of its biodegradation (Table 2). The single electrospun PCL scaffold has not shown any weight-loss ability, even after 60 days of PBS incubation. This is explained by the low permeability of the scaffold due to its highly hydrophobic nature. The degradation process of an implanted scaffold begins when the material starts interacting with the tissue fluids, thus adsorbing water and changing its structure to promote the hydrolysis process. The material’s hydrophilicity is not influential in the case of enzymatic degradation [50,51]. The result of the single electrospun PCL is in accordance with another reported study, where the electrospun PCL showed insignificant weight loss of 1.44% after 90 days of PBS incubation. An opposite effect of the same scaffold was observed in the case of PBS/lipase medium. Thus, the enzymatic degradation of the PCL was 97.11% after 90 days of incubation [24]. As reported, the degradation rate of PCL electrospun scaffolds can be tailored through its combination with other biomaterials (e.g., PCL/gelatin plus cellulose nanocrystals) [52], or through the design of different configurations, i.e., core/shell fibers, layered structures, and so forth.

Human LSCs were successfully cultivated on both electrospun PCL and PCL/anti-VEGF scaffolds, as first confirmed by the SEM (Figure 9) and the confocal images (Figure 10). LSC coverage was more evident in case of the biologically active scaffold, as shown in Figure 10B,B1, where the cells can be identified in more detailed conformations. The identification of the cultured LSCs in terms of stem cell presence (cells positive on p63 marker) or cell corneal epithelial differentiation (cells positive on CK3 marker) is essential as the success of a transplant in corneal tissue repair is reported to be in case of more than 3% of detected p63-bright cells [53]. The confocal-identified p63- and CK3-positive LSCs are in accordance with the SEM observations, whereby the PCL/Anti-VEGF scaffolds showed to be more favorable for the adhesion of the cultured cells, which means the same can support further growth and proliferation of the LSCs. The results confirmed the advantageous effect of the biological component on cell life, which is a dominant factor in damaged tissue regeneration.

The limitation of the current work concerns the statistical analysis of the anti-VEGF release profile, the evaluation of its bioactivity maintenance, and cell proliferation analysis restricted by the availability of human donor LSCs that would further confirm the biocompatibility of the PCL/Anti-VEGF scaffolds. Thus, future research will explore the anti-VEGF function sustainability, in terms of its in-vitro release and long-term bioactivity, as well as the quantitative analysis of viable LSCs adhered on the scaffolds, including both stem cells positive on the p63 marker or differentiated corneal epithelial cells positive on the CK3 marker.

## 5. Conclusions

To prevent uncontrollable ocular vascularization, VEGF blocking agents are incorporated into engineered biomaterials to support their function and sustained target release. This study considered the design of electrospun scaffolds based on PCL treated with anti-VEGF through simple physical adsorption. The aim was to evaluate the PCL/anti-VEGF scaffold reliability in terms of its physicochemical function to support LSCs for corneal repair. The addition of the biological component influenced PCL scaffold morphology as it resulted in fiber diameter and pore area increases by ~24% and ~82%, respectively, while it slightly reduced its total porosity due to the closure of microfibrous structure voids. The anti-VEGF had a positive effect on the PCL wetting ability and its tensile property, as it fully reduced its hydrophobicity and increased its Young modulus by almost three-fold at both strains of 5 and 10%. The gradual weight loss of the scaffold was significantly beneficial as the biodegradation rate completely increased to ~36% after 60 days of PBS incubation, which was in accordance with its sustained agent delivery (total of 30%) after Day 4 of PBS incubation. The results further confirmed PCL/anti-VEGF greater biocompatibility compared to a single PCL, which showed to be more favorable to LSC adhesion, growth, and differentiation as observed by the SEM, and p63 (stem cells presence) and CK3 (cell corneal epithelial differentiation) marker identification after cells staining. The results will contribute to the design of electrospun scaffolds for sustained anti-VEGF delivery to inhibit angiogenesis in the process of LSC ocular tissue engineering. To overcome the limitations of the current study, future research will focus on the evaluation of the biological compound sustained bioactivity, its release pace statistics, and cell proliferation quantitative analysis through the identification of p63 and CK3 positive stem cells or differentiated corneal epithelial cells, respectively.

## Figures and Tables

**Table 1:** Maximum force, elongation at break and tensile strength of the electrospun scaffolds.

Electrospun Scaffolds	F_max_ (N)	ɛ (%)	σ (N/mm^2^)
18% PCL	3.84 ± 0.30	181.85 ± 21.65	2.31 ± 0.18
18% PCL/anti-VEGF	5.43 ± 0.88	99.00 ± 18.19	1.41 ± 0.14

**Table 2:** Biodegradation of the electrospun scaffolds after incubation in PBS solution.

Electrospun Scaffolds	7 Days of Incubation	60 Days of Incubation
	Weight Loss (%)	
18% PCL	negative	negative
18% PCL/Anti-VEGF	13.49 ± 5.69	35.89 ± 1.69

## Data Availability

Not applicable.
